# Supplementary material for: The causal relationship between the human gut microbiota and pyogenic arthritis: a Mendelian randomization study
Source: Front Cell Infect Microbiol. 2024 Nov 26;14:1452480. doi: 10.3389/fcimb.2024.1452480 (PMC11629706; doi:10.3389/fcimb.2024.1452480)
Supplement: Supplementary file 1 [file DataSheet1.zip › mendelian test/ebi-a-GCST90027670.csv_forest.pdf]

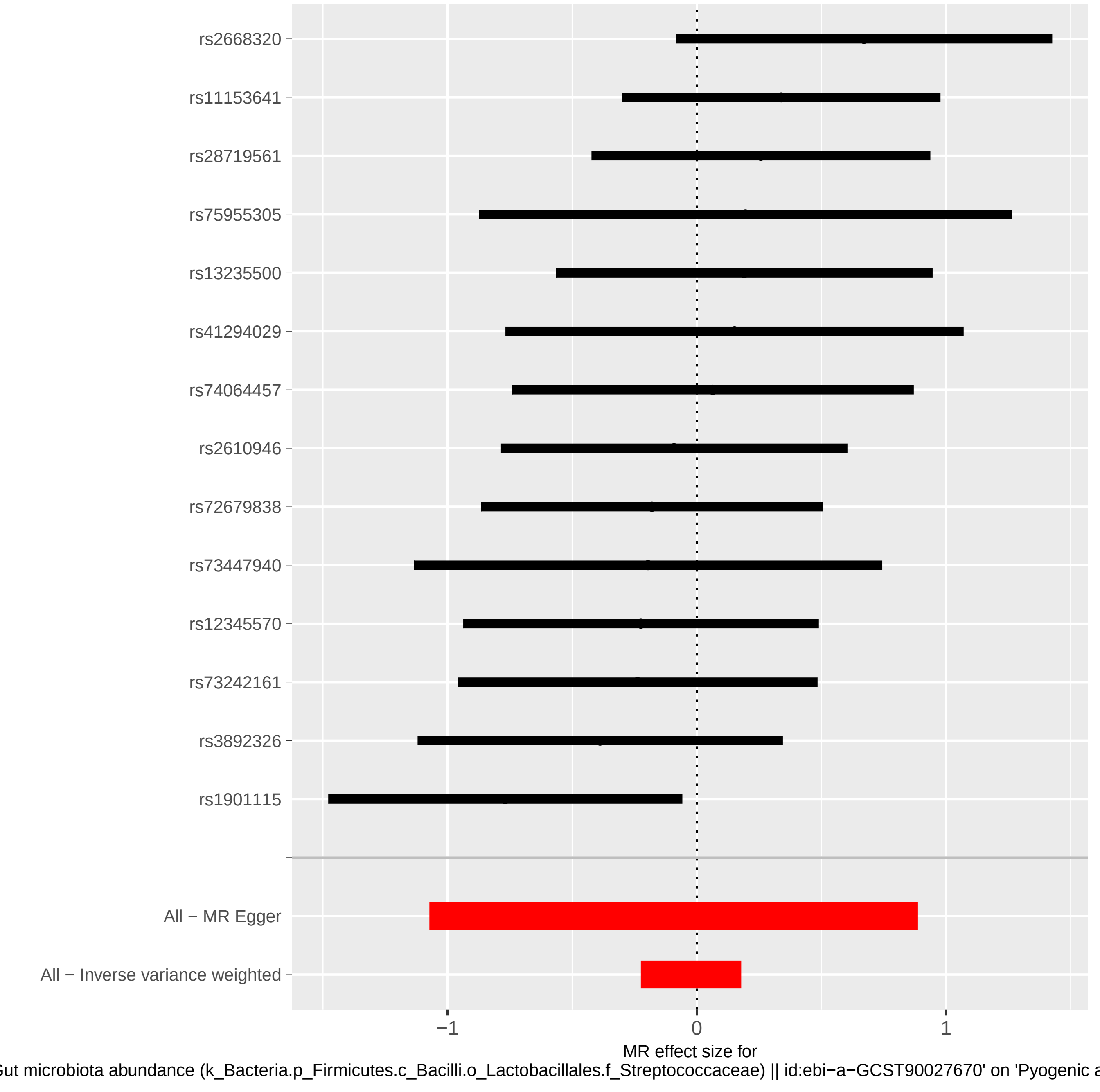
rs2668320
rs11153641
rs28719561
rs75955305
rs13235500
rs41294029
rs74064457
rs2610946
rs72679838
rs73447940
rs12345570
rs73242161
rs3892326
rs1901115
All − MR Egger
All − Inverse variance weighted
−1
0
1
MR effect size for
Gut microbiota abundance (k_Bacteria.p_Firmicutes.c_Bacilli.o_Lactobacillales.f_Streptococcaceae) || id:ebi−a−GCST90027670' on 'Pyogenic a
